# Exercise as a Metabolic Therapy for MASLD: Beyond Weight Loss Toward Sustainable Exercise Strategies

**DOI:** 10.3390/medicina62040784

**Published:** 2026-04-18

**Authors:** Hee-Tae Roh, Ju-Yong Bae

**Affiliations:** 1 Department of Sports Science, College of Arts and Sports, Sun Moon University, Asan-si 31460, Republic of Korea; smuhtroh@sunmoon.ac.kr; 2 Department of Physical Education, College of Arts and Sports, Dong-A University, Busan 49315, Republic of Korea

**Keywords:** metabolic dysfunction–associated steatotic liver disease (MASLD), exercise therapy, aerobic exercise, resistance training, hepatic steatosis, insulin resistance, metabolic adaptation

## Abstract

Metabolic dysfunction–associated steatotic liver disease (MASLD) is a systemic metabolic disorder characterized by impaired metabolic flexibility involving the liver, skeletal muscle, and adipose tissue. Although weight loss has traditionally been emphasized in its management, emerging evidence suggests that exercise exerts therapeutic effects beyond body weight reduction. This narrative review aims to examine exercise as a metabolic therapy for MASLD by integrating mechanistic insights and clinical evidence. Exercise improves hepatic steatosis, insulin resistance, mitochondrial function, and inflammatory signaling through interconnected pathways, including activation of AMPK-related signaling, enhanced fatty acid oxidation, and muscle–liver crosstalk mediated by myokines. Importantly, these benefits can occur independently of weight loss, supporting a shift from weight-centered to metabolism-focused treatment strategies. Both aerobic and resistance exercise demonstrate efficacy, with combined approaches providing complementary benefits. In conclusion, exercise should be considered a central therapeutic strategy for MASLD by restoring metabolic flexibility rather than solely promoting weight reduction. Future research should focus on optimizing individualized and sustainable exercise prescriptions to enhance long-term clinical outcomes.

## 1. Introduction

Metabolic dysfunction–associated steatotic liver disease (MASLD) is defined as hepatic steatosis in the presence of at least one cardiometabolic risk factor (e.g., overweight/obesity, type 2 diabetes, or metabolic dysregulation), according to an international consensus panel [1]. Recent epidemiological estimates suggest that MASLD affects approximately 38% of adults and 7–14% of children and adolescents globally [2]. MASLD is increasingly recognized not merely as a liver-specific disorder but as a systemic metabolic disease characterized by insulin resistance and closely associated with an elevated risk of type 2 diabetes and atherosclerotic cardiovascular disease [3].

Importantly, MASLD is recognized as a heterogeneous disease spectrum with varying degrees of severity, including steatosis, metabolic dysfunction–associated steatohepatitis (MASH), fibrosis, and hepatocellular carcinoma (HCC) [4,5]. This spectrum of disease severity highlights the clinical importance of metabolic interventions, particularly in preventing advanced liver disease [6].

The terminology of nonalcoholic fatty liver disease (NAFLD) has recently been updated to MASLD to better reflect its underlying metabolic etiology [1]. This shift highlights the central role of metabolic dysfunction, including insulin resistance and obesity, in the development of hepatic steatosis [7,8]. Accordingly, MASLD should be understood as a disorder of dysregulated lipid and glucose metabolism, underscoring the importance of mechanistic approaches targeting systemic metabolism [9].

Current clinical guidelines recommend a reduction of approximately 5–10% of initial body weight as a primary therapeutic target for MASLD management because such weight loss is associated with reductions in hepatic fat accumulation and improvements in liver histology [6,10,11]. However, achieving and maintaining meaningful weight loss in real-world clinical settings remains challenging, and adherence to long-term lifestyle interventions is often limited [12]. In addition, individuals with sarcopenic obesity may be at a particular disadvantage, as weight loss–focused interventions can further reduce skeletal muscle mass and exacerbate metabolic dysfunction [13,14]. This highlights the need for therapeutic strategies that preserve skeletal muscle while improving metabolic health.

Growing evidence indicates that exercise can improve hepatic steatosis and metabolic function even in the absence of substantial weight loss. Both aerobic and resistance exercise have been shown to reduce hepatic lipid accumulation and improve metabolic markers independent of body weight changes [15,16]. These findings suggest that the therapeutic benefits of exercise extend beyond simple reductions in body weight and may instead be mediated by exercise-induced metabolic adaptations, including improved mitochondrial function and modulation of the skeletal muscle–liver axis [17].

While exercise has demonstrated clear benefits in improving hepatic steatosis and metabolic dysfunction, the management of MASH remains more complex due to the presence of inflammation and fibrosis [18,19]. Accordingly, recent advances in pharmacological therapy include the clinical use of glucagon-like peptide-1 (GLP-1) receptor agonists (e.g., semaglutide) and the recent approval of Resmetirom as a targeted treatment for MASH [20,21], underscoring the need for integrated therapeutic strategies.

Therefore, this review examines the relative roles of aerobic and resistance exercise in MASLD, discusses the metabolic mechanisms through which exercise improves hepatic steatosis independent of weight loss, and highlights practical considerations and future directions for exercise-based interventions in MASLD management.

## 2. Pathophysiological Mechanisms of MASLD

MASLD is characterized not only by hepatic lipid accumulation but also by systemic metabolic dysregulation, in which the balance among fatty acid influx, synthesis, oxidation, and export becomes disrupted [22]. Under physiological conditions, hepatic lipid homeostasis is tightly regulated; however, persistent metabolic disturbances such as excessive caloric intake, physical inactivity, obesity, and insulin resistance promote hepatic triglyceride accumulation [23]. A major contributor to hepatic steatosis is the increased delivery of free fatty acids (FFAs) from adipose tissue, together with enhanced hepatic de novo lipogenesis. This increase in de novo lipogenesis should be interpreted not only as a liver-specific process but also as a consequence of systemic substrate overflow, in which reduced glucose utilization in skeletal muscle and impaired lipid buffering in adipose tissue drive excess carbohydrate and lipid flux toward the liver [24,25]. Concurrently, fatty acid oxidation is impaired due to reduced peroxisome proliferator-activated receptor-α (PPARα) signaling and mitochondrial dysfunction, further exacerbating lipid accumulation within hepatocytes [26,27,28,29].

Importantly, hepatic lipid accumulation in MASLD should be understood within the context of whole-body metabolic regulation rather than liver-specific processes alone. In insulin-resistant states, impaired suppression of adipose tissue lipolysis leads to elevated circulating FFAs, which are subsequently taken up by the liver and contribute to triglyceride synthesis [30]. Hepatic fatty acid export occurs primarily through the synthesis and secretion of very low-density lipoproteins (VLDLs); however, in insulin-resistant conditions, this export becomes relatively insufficient. This is not necessarily due to an absolute reduction in VLDL secretion, but rather because hepatic lipid influx and de novo lipogenesis exceed the liver’s capacity for VLDL assembly and export [9,31]. Consequently, a mismatch between lipid input and output develops under conditions of excessive substrate availability, promoting triglyceride retention within hepatocytes. This imbalance is further associated with impaired mitochondrial fatty acid oxidation and reduced metabolic flexibility, ultimately exacerbating hepatic lipid accumulation [32]. In addition, compensatory hyperinsulinemia contributes to hepatic steatosis. Due to increased insulin exposure via the portal circulation, the liver develops selective insulin resistance, characterized by impaired suppression of gluconeogenesis but preserved lipogenesis, thereby further promoting hepatic lipid accumulation [33].

Beyond hepatic processes, inter-organ metabolic interactions play a critical role in MASLD pathogenesis. Skeletal muscle represents a major site of glucose and fatty acid utilization and serves as a key metabolic sink for circulating substrates [34]. Reduced muscle oxidative capacity, commonly associated with physical inactivity and insulin resistance, has been demonstrated in both human studies and experimental models, and contributes to metabolic substrate overflow toward the liver [17,34,35]. In addition, hormonal and regulatory factors, including insulin signaling and lipid partitioning processes, influence the distribution of circulating lipids among tissues, further modulating hepatic lipid burden [36]. This suggests that impaired skeletal muscle metabolism is not merely a consequence of systemic metabolic dysfunction but a key driver of hepatic lipid accumulation [34,36].

From a whole-body perspective, MASLD can therefore be understood as a disorder of dysregulated substrate flux among adipose tissue, skeletal muscle, and liver. When energy demand in skeletal muscle is low, substrate utilization declines, and excess fatty acids are redirected toward the liver, promoting steatosis. Conversely, conditions that increase skeletal muscle energy demand enhance fatty acid oxidation and glucose uptake in peripheral tissues, thereby reducing substrate delivery to the liver. This adipose–liver–muscle axis provides a mechanistic framework linking systemic metabolic dysfunction to hepatic lipid accumulation.

Persistent hepatic lipid accumulation further aggravates mitochondrial dysfunction and increases reactive oxygen species production, leading to oxidative stress [37]. This process activates inflammatory signaling pathways, including nuclear factor-κB, and promotes the release of pro-inflammatory cytokines such as tumor necrosis factor-α and interleukin-1β. Although interleukin-6 is also involved in MASLD pathophysiology, its role is more complex and context-dependent, as chronic elevation may contribute to metabolic dysfunction, whereas exercise-induced skeletal-muscle-derived IL-6 functions as a myokine that exerts anti-inflammatory effects and enhances lipid metabolism in peripheral tissues [38,39]. In this inflammatory context, transforming growth factor-β (TGF-β) acts as a central profibrotic mediator that drives extracellular matrix remodeling and collagen deposition, thereby contributing to the progression of hepatic fibrosis [40,41]. Collectively, MASLD is now recognized as a complex metabolic disorder involving dysregulated lipid flux, impaired mitochondrial function, oxidative stress, and chronic inflammation [42].

These pathophysiological characteristics provide key targets for exercise-based interventions. Evidence from animal and mechanistic studies indicates that exercise activates AMP-activated protein kinase (AMPK) in both skeletal muscle and hepatocytes, thereby suppressing hepatic lipogenesis, enhancing mitochondrial biogenesis, and promoting fatty acid oxidation while improving systemic insulin sensitivity [43,44]. AMPK suppresses hepatic lipogenesis partly through inhibition of acetyl-CoA carboxylase (ACC). Notably, ACC exists as two isoforms, ACC1 and ACC2, which play distinct roles: ACC1 primarily regulates de novo lipogenesis, whereas ACC2 regulates fatty acid oxidation by modulating mitochondrial fatty acid entry [45]. In addition, increased energy demand in skeletal muscle during exercise enhances peripheral glucose uptake and fatty acid utilization, thereby redistributing metabolic substrates away from the liver. This facilitates peripheral lipid clearance and improves substrate partitioning across tissues, thereby reducing lipid overflow to the liver. Experimental studies have demonstrated that exercise alone, even without dietary modification, can reduce hepatic lipid accumulation and improve metabolic markers independent of body weight changes. Furthermore, both aerobic and resistance exercise have been shown to improve hepatic lipid metabolism in obese animal models, accompanied by increased hepatic AMPK phosphorylation and reduced hepatic triglyceride levels [46,47]. These findings indicate that exercise functions not merely as a weight-loss strategy but as a systemic metabolic regulator capable of modulating inter-organ substrate flux and the underlying mechanisms of MASLD.

## 3. Aerobic Exercise and MASLD

Aerobic exercise is widely recognized as an effective intervention for improving hepatic steatosis and systemic insulin sensitivity in MASLD [48,49,50]. Moderate-to-vigorous aerobic exercise has been shown in experimental studies to activate AMPK, which suppresses hepatic lipogenesis while stimulating the expression of peroxisome proliferator-activated receptor gamma coactivator-1α (PGC-1α). This signaling pathway enhances mitochondrial biogenesis and oxidative capacity, thereby promoting fatty acid β-oxidation and contributing to reductions in hepatic triglyceride accumulation [51,52].

In addition, AMPK activation phosphorylates ACC, reducing malonyl-CoA levels and facilitating mitochondrial fatty acid transport via carnitine palmitoyltransferase-1, ultimately enhancing fatty acid oxidation [53,54]. Experimental studies in high-fat-diet-induced obese models have consistently demonstrated increased hepatic AMPK activation and upregulation of genes associated with fatty acid oxidation following aerobic exercise [46].

Clinical evidence further supports these findings. Supervised aerobic training performed for approximately 45–60 min per session, three to five times per week, has been shown to significantly reduce hepatic fat content measured by proton magnetic resonance spectroscopy, even in the absence of substantial weight loss [49]. Similarly, structured exercise programs conducted for 8–16 weeks can produce clinically meaningful reductions in hepatic steatosis independent of body weight changes [50].

From a metabolic perspective, aerobic exercise improves MASLD primarily by enhancing oxidative capacity in skeletal muscle and liver, thereby increasing fatty acid utilization and reducing hepatic lipid accumulation [55,56,57]. However, long-term adherence to aerobic exercise programs may be challenging in certain populations, highlighting the need for complementary exercise strategies [58,59].

## 4. Resistance Exercise and MASLD

Resistance exercise represents an important therapeutic strategy for MASLD and should not be considered merely an alternative to aerobic exercise. Resistance training increases skeletal muscle mass and improves muscle function, thereby enhancing basal metabolic rate and insulin-mediated glucose utilization [60,61]. Rather than primarily increasing fat oxidation, resistance exercise expands the metabolic capacity of peripheral tissues to store and utilize substrates, indirectly reducing substrate overload delivered to the liver [62,63].

In addition to these systemic effects, resistance exercise induces molecular adaptations through the skeletal muscle–liver axis. Experimental studies suggest that resistance exercise modulates hepatic lipid metabolism through increased expression of lipolytic enzymes and activation of pathways associated with fatty acid oxidation [46,47].

Resistance exercise promotes increases in skeletal muscle mass and induces the secretion of various myokines, which play an important role in regulating systemic metabolic function and energy metabolism [64,65,66]. In particular, irisin has been implicated in lipid metabolic regulation; mechanistic studies have shown that recombinant irisin reduces lipogenesis in hepatocytes, accompanied by decreased expression of sterol regulatory element-binding protein-1c (SREBP-1c) and downstream lipogenic enzymes such as fatty acid synthase [67]. In both animal and human studies, exercise has been shown to modulate circulating fibroblast growth factor 21 (FGF21) levels [68,69]. However, it is important to note that most evidence regarding FGF21 responses has been derived from studies on endurance exercise rather than resistance training. FGF21 contributes to systemic energy homeostasis by regulating lipid and glucose metabolism, enhancing fatty acid oxidation, and facilitating adaptive responses to metabolic stress [70,71]. In addition, FGF21 has been reported to promote thermogenic programming through the induction of uncoupling proteins, enhance lipid uptake and storage in adipose tissue, and modulate macronutrient preference, thereby contributing to reduced ectopic lipid accumulation in the liver [70]. These metabolic effects are considered relevant in the context of MASLD/MASH, where FGF21 has emerged as a potential therapeutic target [71]. Accordingly, these responses are not uniform, with reports of both increases and decreases depending on exercise modality, duration, and metabolic status, suggesting that the role of FGF21 in MASLD/MASH is complex and context-dependent [72,73].

Clinical intervention studies support these mechanistic findings. Resistance training performed three times per week for approximately 8–12 weeks has been shown to significantly reduce hepatic fat content in individuals with MASLD, even in the absence of significant weight loss [74,75].

From a metabolic perspective, resistance exercise improves MASLD primarily by increasing skeletal muscle mass and expanding peripheral capacity for glucose and lipid utilization [62,76]. Increased muscle mass acts as a major metabolic sink for circulating substrates, thereby reducing the metabolic burden placed on the liver [63]. These adaptations indicate that resistance exercise improves hepatic steatosis through mechanisms that extend beyond simple energy expenditure. In addition to these metabolic effects, exercise may also influence energy intake and appetite regulation. Both acute and chronic exercise can modulate appetite-related hormones, such as ghrelin and glucagon-like peptide-1 (GLP-1), thereby affecting satiety and subsequent food intake [77].

## 5. Integrated Effects of Exercise on MASLD

Both aerobic and resistance exercise exert beneficial effects on hepatic lipid metabolism, insulin sensitivity, and mitochondrial function through overlapping mechanisms [36,78]. These shared effects are mediated by improved substrate utilization, enhanced fatty acid oxidation, and increased peripheral glucose uptake [35,79].

While aerobic exercise primarily enhances oxidative capacity and energy expenditure, resistance exercise contributes to increased skeletal muscle mass and expansion of metabolic storage capacity, although both modalities improve hepatic fat content and insulin resistance in MASLD [50,80]. However, these distinctions should be viewed as complementary rather than independent, as both exercise modalities contribute to systemic metabolic improvements relevant to MASLD [34,36].

Importantly, emerging evidence suggests that combined aerobic and resistance exercise may provide additive or synergistic benefits. By simultaneously enhancing oxidative metabolism and increasing skeletal muscle mass, combined training may improve whole-body substrate utilization and reduce excess substrate delivery to the liver [81]. This integrated metabolic adaptation may be particularly effective in attenuating hepatic steatosis and improving insulin sensitivity [82]. Furthermore, combined exercise interventions may improve long-term adherence and functional capacity, particularly in populations with metabolic disorders or sarcopenia [83,84]. Therefore, aerobic and resistance exercise should be considered complementary components of a comprehensive exercise strategy for MASLD rather than isolated modalities [85].

**Figure 1:**
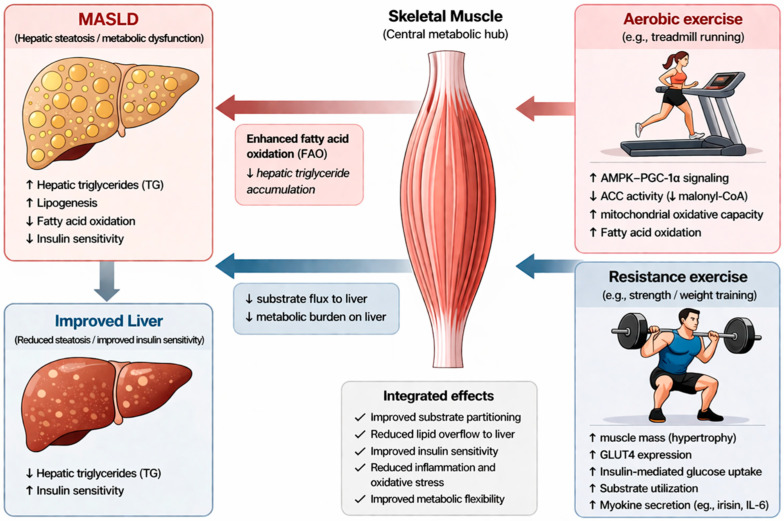
Complementary Metabolic Mechanisms of Exercise in MASLD. Upward arrows (↑) indicate an increase, whereas downward arrows (↓) indicate a decrease.

Furthermore, accumulating evidence indicates that exercise exerts beneficial effects beyond hepatic steatosis by modulating inflammation and oxidative stress. In animal models of MASLD, both aerobic and resistance exercise have been shown to attenuate hepatic inflammation and improve oxidative stress status [79,86,87,88,89]. Consistent with these findings, human studies have demonstrated that exercise interventions reduce systemic and hepatic inflammatory markers and improve liver-related outcomes [90,91,92,93]. Importantly, evidence from biopsy-based studies further suggests that exercise may improve histological features such as hepatocyte ballooning and lobular inflammation, although its effects on fibrosis remain limited and inconsistent [94]. Collectively, these findings indicate that the therapeutic effects of exercise extend beyond reductions in hepatic fat content to include improvements in inflammation, oxidative stress, and potentially fibrosis, highlighting its role as a comprehensive metabolic intervention in MASLD (Figure 1).

This schematic illustrates the mechanistic pathways through which aerobic and resistance exercise improve hepatic steatosis and metabolic dysfunction independent of weight loss, with skeletal muscle acting as a central metabolic hub regulating inter-organ substrate partitioning [34,36]. Aerobic exercise primarily activates AMP-activated protein kinase (AMPK) and its downstream signaling pathways, including PGC-1α, leading to enhanced mitochondrial oxidative capacity and increased fatty acid oxidation (FAO) [43,51,52]. In addition, AMPK-mediated inhibition of acetyl-CoA carboxylase (ACC) reduces malonyl-CoA levels and suppresses de novo lipogenesis while facilitating mitochondrial fatty acid transport [53,54]. These coordinated adaptations contribute to a reduction in intrahepatic triglyceride accumulation [46,49]. In contrast, resistance exercise predominantly induces skeletal muscle hypertrophy and increases GLUT4 expression, thereby enhancing insulin-mediated glucose uptake and peripheral substrate utilization [60,61]. These adaptations expand the metabolic capacity of skeletal muscle, resulting in reduced flux of circulating substrates, particularly free fatty acids and glucose, to the liver and consequently lowering hepatic metabolic burden [62,63]. Skeletal muscle plays a central role in systemic metabolic regulation, and impaired muscle oxidative capacity contributes to substrate overflow toward the liver in insulin-resistant states [34,35]. Exercise-induced improvements in muscle function facilitate redistribution of metabolic substrates away from the liver, thereby attenuating lipid overflow and improving hepatic lipid homeostasis [36,55]. Collectively, both exercise modalities converge to improve hepatic steatosis and insulin sensitivity through complementary mechanisms involving increased fatty acid oxidation, reduced substrate delivery to the liver, and improved metabolic flexibility [50,78,79].

## 6. Weight Loss and MASLD: Expanding the Therapeutic Perspective

Aerobic and resistance exercise influence metabolic pathways involved in MASLD through distinct mechanisms [48,52,95]. Aerobic exercise primarily enhances mitochondrial oxidative capacity through activation of the AMPK–PGC-1α signaling pathway, thereby promoting fatty acid β-oxidation [96]. In contrast, resistance exercise improves skeletal muscle mass and insulin-mediated glucose utilization, reducing the metabolic substrate load delivered to the liver [60,97]. From this perspective, aerobic exercise can be understood as a strategy that enhances the capacity to oxidize lipids, whereas resistance exercise expands the peripheral tissue’s capacity to process metabolic substrates. Despite these mechanistic differences, both exercise modalities ultimately converge on improving hepatic lipid metabolism and correcting the imbalance between lipid synthesis and degradation [50,98]. This convergence suggests that aerobic and resistance exercise should be viewed not as competing interventions but as complementary strategies in MASLD management.

Current clinical guidelines continue to emphasize weight loss as a central therapeutic goal in MASLD. The American Association for the Study of Liver Diseases (AASLD) practice guidance indicates that a weight reduction of approximately 3–5% of initial body weight can reduce hepatic steatosis, while weight loss of 7–10% may lead to improvements in inflammation and resolution of nonalcoholic steatohepatitis [99]. Greater weight reductions of more than 10% have been associated with improvements in liver fibrosis, suggesting a dose–response relationship between weight loss and histological improvement [99]. Similarly, the Asia-Pacific Working Party on NAFLD recommends a minimum weight loss of approximately 5% to reduce hepatic fat, with greater reductions of 7–10% associated with improved liver histology [100]. While these thresholds are widely used in clinical practice, emerging evidence suggests that even modest weight reductions of approximately 1–3% may contribute to improvements in metabolic parameters and hepatic lipid metabolism, although the magnitude of these effects is generally smaller than that observed with greater weight loss [101,102].

However, achieving and maintaining substantial weight loss in real-world clinical settings remains challenging. Long-term weight maintenance after lifestyle intervention is difficult, and weight regain is commonly observed following initial reductions [103]. Moreover, in older individuals or patients with sarcopenic obesity, excessive weight loss may lead to reductions in skeletal muscle mass, potentially worsening functional outcomes [104].

In contrast, an increasing number of studies have demonstrated that exercise interventions can significantly reduce hepatic fat even in the absence of substantial weight loss [47,105,106,107]. Randomized controlled trials and meta-analyses have reported that structured exercise training can reduce hepatic fat content measured by magnetic resonance imaging by more than 30%, often with only minimal changes in body weight [105]. Other clinical studies have similarly reported improvements in liver fat and liver stiffness following exercise programs lasting approximately three months, even when overall body weight remains relatively stable [106].

However, these findings should be interpreted with caution. Even modest reductions in body weight or fat mass may contribute to improvements in hepatic lipid metabolism [99,100], and it is often difficult to fully disentangle the independent effects of exercise from concurrent changes in body composition. For example, some intervention studies have reported significant time effects on BMI and fat mass despite relatively small absolute weight changes [50], suggesting that alterations in substrate flux, including lipid and glucose metabolism, may partially mediate the observed benefits.

Taken together, these findings suggest that although exercise-induced improvements in hepatic steatosis are not solely explained by weight loss [99,100,108], both exercise and changes in body composition likely contribute to the overall metabolic response. Accordingly, exercise should be recognized as a key metabolic intervention [109], while acknowledging the complex interaction between physical activity, body composition, and hepatic lipid metabolism. Recent advances in pharmacological therapies have introduced highly effective anti-obesity agents that facilitate substantial weight loss and metabolic improvement [110,111]. In this context, future research is warranted to explore how exercise and pharmacological approaches can be effectively integrated to optimize metabolic and hepatic outcomes in MASLD. Importantly, this interaction is complex, as both exercise and pharmacological agents can influence energy balance through distinct mechanisms. While exercise increases energy expenditure and may modulate appetite regulation, pharmacological therapies—particularly those targeting appetite pathways—can reduce energy intake [77,112]. Therefore, integrated strategies should consider both energy expenditure and intake to achieve optimal outcomes.

## 7. Toward Sustainable Exercise-Based Interventions

As discussed above, the metabolic benefits of exercise in MASLD arise not merely from energy expenditure but from direct regulation of hepatic lipid metabolism and systemic insulin sensitivity. Consequently, when considering optimal exercise interventions for MASLD, focusing solely on identifying the most effective exercise modality may be overly simplistic [79,95]. Instead, the key consideration is whether a particular exercise strategy can induce stable metabolic adaptations and whether these adaptations can be maintained over the long term.

Aerobic exercise improves oxidative capacity in both skeletal muscle and liver, thereby enhancing fatty acid oxidation, whereas resistance exercise increases skeletal muscle mass and expands the peripheral capacity for substrate utilization. Through these distinct mechanisms, both exercise modalities ultimately regulate the same pathophysiological targets, including hepatic lipid accumulation and systemic metabolic dysfunction [17,74]. Therefore, from a clinical perspective, it may be more appropriate to adopt flexible exercise strategies tailored to individual characteristics rather than rigidly prescribing a single exercise modality. Factors such as age, baseline physical fitness, comorbidities, and individual exercise tolerance should be considered when designing exercise interventions [99,100,113].

For example, patients with sarcopenic obesity or reduced muscle strength may benefit from resistance training aimed at preserving or increasing skeletal muscle mass [114]. Conversely, individuals with low cardiorespiratory fitness may initially benefit from gradually progressive aerobic exercise designed to improve oxidative metabolic capacity [115].

Ultimately, the most important determinant of therapeutic success is not short-term exercise intensity or caloric expenditure but the long-term sustainability of exercise behavior. Exercise programs that can be consistently maintained within daily life are more likely to produce durable metabolic benefits and sustained reductions in hepatic fat accumulation. From this perspective, exercise prescriptions for MASLD should be understood not simply as tools for weight reduction but as long-term strategies for metabolic reprogramming.

## 8. Practical Considerations for Exercise Prescription in MASLD

Exercise interventions for MASLD require a strategic design that reflects both the pathophysiological characteristics of the disease and the functional capacity of individual patients. Current physical activity guidelines recommend at least 150–300 min per week of moderate-intensity aerobic exercise or 75–150 min per week of vigorous-intensity activity, corresponding to approximately 500–1000 MET-minutes per week, together with resistance training performed two to three times per week [116,117]. However, exercise prescriptions for MASLD should not be defined solely by exercise dose. Evidence indicates that exercise can reduce hepatic fat content even in the absence of significant body weight loss [105], suggesting that the primary therapeutic objective of exercise is to induce favorable metabolic adaptations rather than weight reduction itself. Accordingly, promoting sustainable exercise behaviors that induce long-term metabolic adaptations may be more important than focusing solely on short-term increases in exercise volume when designing effective exercise interventions for MASLD.

First, aerobic and resistance exercise should be considered complementary rather than mutually exclusive strategies. Both exercise modalities have been shown to reduce hepatic fat content, and comparative studies have generally failed to demonstrate a clear superiority of one modality over the other [118]. Aerobic exercise primarily improves oxidative metabolism and fatty acid utilization, whereas resistance exercise increases skeletal muscle mass and enhances insulin-mediated glucose uptake. Combining these two exercise modalities may therefore provide synergistic benefits by simultaneously improving oxidative capacity and muscle-based metabolic function.

Second, exercise prescriptions should be individualized according to patient characteristics, including age, muscle strength, cardiorespiratory fitness, and the presence of comorbid conditions. In older adults or individuals with sarcopenic obesity, incorporating resistance training may be particularly important to preserve skeletal muscle mass and metabolic function. Conversely, individuals with low baseline aerobic capacity may benefit from gradually progressive aerobic training aimed at improving cardiorespiratory fitness and mitochondrial oxidative capacity. Such individualized approaches may enhance adherence and play an important role in maintaining long-term metabolic improvements [119].

Third, evaluating the effectiveness of exercise interventions solely on the basis of body weight change may be insufficient. Improvements in body composition, insulin sensitivity, and hepatic lipid metabolism can occur even when overall body weight remains relatively unchanged [74]. Therefore, a comprehensive assessment including body composition, metabolic markers, and imaging-based liver fat measurements should be considered when evaluating therapeutic outcomes.

Lastly, the long-term success of exercise interventions depends not only on program design but also on behavioral and environmental factors that support sustained adherence. Exercise programs that can be integrated into daily routines and supported by behavioral interventions are more likely to produce lasting metabolic benefits and sustained improvements in hepatic metabolic health.

Despite the well-established benefits of exercise, potential risks and practical challenges should also be considered when prescribing exercise for individuals with MASLD. Individuals who are physically inactive or have low physical fitness may be at a higher risk of musculoskeletal injury when initiating exercise, particularly during the early stages of training [120]. Accordingly, patients with reduced physical capacity, including those with sarcopenic obesity or those newly initiating exercise, require particular caution. Therefore, appropriate pre-exercise assessment and adherence to established exercise prescription guidelines, with gradual progression in intensity and volume, are essential to ensure safe and individualized exercise implementation [121].

In addition, barriers such as low motivation, comorbid conditions, physical limitations, and limited access to structured exercise programs may hinder long-term adherence to exercise interventions [122]. Therefore, exercise prescriptions should be carefully tailored to individual capacity, considering disease severity, baseline physical fitness, and the presence of comorbid conditions, with gradual progression in intensity and volume.

Importantly, therapeutic goals may differ depending on patient characteristics. While improving hepatic steatosis may be an initial target, other clinically meaningful outcomes, such as reducing inflammation, preventing fibrosis progression, improving physical function, and enhancing long-term adherence, should also be considered when designing individualized exercise interventions. These priorities are supported by current clinical guidelines and evidence from both imaging-based and biopsy-based studies, emphasizing the importance of targeting disease progression beyond simple fat reduction [94,99,105,106].

## 9. Future Directions

Future research on MASLD should move beyond the traditional emphasis on weight loss and instead focus on understanding the metabolic adaptations induced by exercise and their temporal dynamics. Although exercise can reduce hepatic fat independent of body weight changes, the precise physiological mechanisms underlying this phenomenon remain incompletely understood. Future studies should aim to clarify the relative contributions of metabolic adaptations such as AMPK activation, mitochondrial biogenesis, and improved skeletal muscle insulin sensitivity to reductions in hepatic fat accumulation.

To achieve this goal, integrated research designs incorporating simultaneous measurements of body weight, body composition, liver fat content, and skeletal muscle metabolic markers will be necessary. Such approaches may provide a more comprehensive understanding of the complex interactions between skeletal muscle metabolism and hepatic lipid regulation.

Another important research direction involves the role of exercise in patients with sarcopenic obesity and MASLD. As global populations age, the coexistence of reduced muscle mass and metabolic liver disease is becoming increasingly common. However, many previous studies have not adequately considered changes in skeletal muscle mass as a key outcome variable [13,123,124]. Investigating the optimal intensity, frequency, and minimum effective dose of resistance exercise for preserving muscle mass while improving hepatic steatosis may therefore provide important insights for clinical practice.

Long-term longitudinal studies are also needed to clarify how exercise-induced metabolic adaptations interact with body weight changes over extended periods. Most existing studies have relatively short intervention durations, typically ranging from several weeks to a few months. Studies with follow-up periods of at least one year may provide valuable insights into the temporal relationship between weight change, body composition, and hepatic fat reduction.

Future studies should explore behavioral and technological strategies that support sustained exercise participation, including supervised and home-based exercise models as well as digital health tools such as wearable activity trackers and remote monitoring systems. Integrating these approaches with metabolic outcome assessments may help develop more effective and sustainable exercise interventions for MASLD.

Finally, the expanding use of pharmacological therapies for MASLD, including GLP-1 receptor agonists and thyroid hormone receptor-β (TRβ) agonists, highlights the need to investigate potential interactions between pharmacological and exercise-based interventions. Understanding whether exercise and pharmacotherapy exert additive or synergistic effects on metabolic flexibility and hepatic fat reduction may help refine future treatment strategies. These agents target distinct metabolic pathways, suggesting that their combination with exercise may produce complementary or synergistic effects.

In this context, it is important to recognize that both pharmacological and lifestyle-based interventions face challenges related to long-term sustainability. While GLP-1 receptor agonists have demonstrated effectiveness in promoting weight loss and improving metabolic outcomes, their primary mechanism involves appetite suppression, delayed gastric emptying, and enhanced central satiety signaling, leading to reduced energy intake. Exercise, in contrast, primarily increases energy expenditure and improves hepatic lipid metabolism and systemic insulin sensitivity. However, pharmacologically induced weight loss may also be accompanied by reductions in lean body mass, highlighting the importance of incorporating resistance exercise to preserve skeletal muscle mass [112,125]. However, as exercise is also a form of lifestyle intervention, maintaining long-term adherence represents a significant challenge.

Therefore, future research should focus not only on the independent effects of exercise and pharmacological treatments but also on integrated strategies that combine these approaches. Such combined interventions may enhance long-term adherence, improve metabolic outcomes, and provide a more sustainable therapeutic framework for individuals with MASLD/MASH.

Beyond these considerations, emerging evidence suggests that circadian regulation plays a critical role in hepatic metabolism and the pathogenesis of MASLD [126]. The liver circadian clock coordinates key metabolic processes, including lipid metabolism, mitochondrial function, and oxidative stress responses [126], and regulates key metabolic genes such as PPARα, PGC-1α, CPT1, and SREBP1 [127]. Disruption of circadian rhythms has been associated with metabolic dysfunction, inflammation, and fibrosis [126,128], with fibrosis being mediated through pathways involving transforming growth factor-β signaling [40].

In this context, the timing of exercise may represent an important but underexplored factor influencing metabolic outcomes. Chrono-exercise strategies, which align physical activity with circadian rhythms, may enhance the metabolic benefits of exercise and improve therapeutic efficacy in MASLD. Future research is warranted to determine the optimal timing and integration of exercise interventions based on circadian biology.

## 10. Limitations of Current Evidence

Despite the growing body of evidence supporting the beneficial role of exercise in MASLD management, several limitations in the current literature should be acknowledged.

First, many studies examining exercise interventions in MASLD have relatively short durations, typically ranging from 8 to 24 weeks. Such short-term interventions may not fully capture the long-term metabolic adaptations associated with sustained exercise behavior.

Second, there is considerable heterogeneity in exercise protocols across studies, including differences in exercise intensity, duration, frequency, and modality. This variability makes it difficult to establish standardized exercise prescriptions for MASLD management.

Third, many studies rely on surrogate markers such as imaging-based assessments of hepatic fat rather than histological outcomes obtained through liver biopsy. Although imaging techniques provide valuable noninvasive measures of hepatic steatosis, they may not fully reflect disease progression or fibrosis development.

Finally, lifestyle intervention studies often involve concurrent dietary changes or increases in overall physical activity outside the formal intervention protocol. These factors may confound the interpretation of the independent effects of structured exercise interventions.

Addressing these limitations through well-controlled, long-term randomized studies will be essential for refining exercise-based therapeutic strategies for MASLD.

## 11. Conclusions

MASLD should be recognized as a systemic disorder characterized by impaired metabolic flexibility across the liver, skeletal muscle, and adipose tissue. In this context, exercise is not merely a supportive lifestyle intervention, but a metabolic therapy that directly targets the underlying pathophysiology.

Evidence consistently shows that exercise improves hepatic steatosis, insulin resistance, and mitochondrial function through integrated mechanisms, including AMPK activation, enhanced fatty acid oxidation, and muscle–liver crosstalk. Notably, these benefits can occur independently of weight loss, highlighting the limitations of weight-centered approaches and supporting a shift toward metabolism-focused strategies.

Clinically, both aerobic and resistance exercise provide meaningful benefits, with combined modalities offering complementary effects. However, individualized prescriptions and long-term adherence remain critical challenges.

In conclusion, exercise should be established as a central therapeutic strategy for MASLD by restoring metabolic flexibility rather than focusing solely on weight reduction. Future approaches should emphasize mechanism-driven, personalized, and sustainable exercise interventions.

## Data Availability

No new data were created or analyzed in this study.
